# Supplementary material for: PTMphinder: an R package for PTM site localization and motif extraction from proteomic datasets
Source: PeerJ. 2019 Jun 4;7:e7046. doi: 10.7717/peerj.7046 (PMC6555389; doi:10.7717/peerj.7046)
Supplement: Table S1 [file peerj-07-7046-s001.docx]

**Supplementary Table 1 - Hypothetical Pseudo-dataset Input Example for phindPTMs Function**

| **Identifier** | **Protein_ID** | **Peptide_Seq** | **Total_Sites** | **PTM_Loc** | **PTM_Score** |
| --- | --- | --- | --- | --- | --- |
| phos_pep1 | Q13131 | tSCGSPNYAAPEVISGR | 1 | T1 | 99.87 |
| phos_pep2 | P17252 | GAENFDKFFt | 1 | T10 | 123 |
| glyc_pep1 | P98160 | SYLSDDEDMLADSISGDDLGsGDLGsGDFQMVYF | 2 | S21;  S26 | 44.2;  56.3 |
| glyc_pep2 | P01583 | YEFILNDALnQSII | 1 | N10 | 20 |
| ubiq_pep1 | P04637 | AHSSHLkSkKGQSTS | 2 | K7;  K9;  K10 | 89.78;  49.75;  49.75 |
| ubiq_pep2 | P46527 | KRPATDDSSTQNK | 2 | K1;  K13 | 99.99;  92.24 |
